# Case report: Kikuchi-Fujimoto disease: unveiling a case of recurrent fever and enlarged cervical lymph nodes in a young female patient with a literature review of the immune mechanism

**DOI:** 10.3389/fimmu.2023.1279592

**Published:** 2024-01-19

**Authors:** Jia-Li Yu, Zhen Li, Bo Zhang, Ya-Nan Huang, Tian-Yu Zhao

**Affiliations:** ^1^ Department of Gastroenterology, The First Affiliated Hospital of Dalian Medical University, Dalian, China; ^2^ Department of Pathology, The First Affiliated Hospital of Dalian Medical University, Dalian, China; ^3^ Department of Infectious Disease, The First Affiliated Hospital of Dalian Medical University, Dalian, China

**Keywords:** Kikuchi-Fujimoto disease, virus-induced inflammation, CD4+ T lymphocytes, CD8+ T lymphocytes, CD123+ plasmacytoid dendritic cells, immune mechanism

## Abstract

The inflammatory response to viral infection is an important component of the antiviral response, a process that involves the activation and proliferation of CD8+ T, CD4+ T, and dendritic cells; thus, viral infection disrupts the immune homeostasis of the organism, leading to an increased release of inflammatory factors. Kikuchi-Fujimoto disease (KFD) is an inflammatory self-limited disorder of unknown etiology, and it is generally believed that the pathogenesis of this disease includes two aspects: viral infection and autoimmune response. Various immune cells, such as CD8+ T lymphocytes, CD4+ T lymphocytes, and CD123+ plasmacytoid dendritic cells, as well as the cytokines they induce and secrete, such as interferons, interleukins, and tumor necrosis factors, play a crucial role in the pathogenesis of KFD. In this article, we present a case study of a young female patient from China who exhibited typical symptoms of lymph node inflammation and fever. The diagnosis of KFD was confirmed through a lymph node biopsy. She presented with elevated ESR, IL-6, and IFN-γ. Viral markers showed elevated IgG and IgM of cytomegalovirus (CMV) and elevated IgG of Epstein–Barr virus (EBV), while changes occurred in the CD4+ T and CD8+ T cell counts. Eventually, the patient achieved disease relief through steroid treatment. Based on these findings, we conducted a comprehensive review of the involvement of viral infection–induced inflammatory response processes and autoimmunity in the pathogenesis of Kikuchi-Fujimoto disease.

## Introduction

The inflammatory response triggered by viral infections plays a crucial role in the antiviral response, which is a process that involves the activation and proliferation of CD8+ T cells, CD4+ T cells, and dendritic cells (1). Consequently, viral infection disrupts the immune homeostasis of the organism, resulting in an increased release of inflammatory factors (2). Kikuchi–Fujimoto disease (KFD), also known as histiocytic necrotizing lymphadenitis, is a self-limiting inflammatory condition with an etiology that remains elusive (3). It is generally believed that the pathogenesis of this disease included two aspects: viral infection and autoimmune response (4). Electron microscopy investigations have shown the presence of tubular reticular structures within the cytoplasm of stimulated lymphocytes and histiocytes in individuals diagnosed with KFD (5).Thus, it is hypothesized that KFD may reflect a self-limited autoimmune condition induced by virus-infected transformed lymphocytes (6, 7). KFD typically presents as an acute to subacute course, characterized by cervical lymph node inflammation and fever, and may involve the skin. Less common manifestations include weight loss, fatigue, night sweats, myalgia, arthralgia, elevated hepatic enzymes, and nervous system involvement, and approximately 15% of patients experience hepatomegaly or splenomegaly (8–10). Sometimes, it occurs in patients with systemic lupus erythematosus (SLE) and can be complicated by macrophage activation syndrome (MAS) (11). Lymph node biopsy is the most recommended method for confirming the diagnosis of Kikuchi-Fujimoto disease.

We report a case of a young female patient from China who presented with typical lymph node enlargement and fever; the diagnosis of KFD was confirmed by lymph node biopsy. Viral markers showed elevated IgG and IgM of cytomegalovirus (CMV) and elevated IgG of Epstein–Barr virus (EBV), while ESR, IL-6, and IFN-γ were elevated. Changes were found in peripheral blood lymphocyte subsets, including CD4+ T and CD8+ T cell counts. Lymph node biopsy pathology showed typical lymph node necrosis, as well as characteristic crescent-shaped nuclei and large immunoblastic cells at the edges of necrotic foci. Immunohistochemistry showed CD68(+), CD163(+), MPO(+), CD123(+) histiocytes surrounding the necrotic areas. Eventually, the patient went into remission with steroid therapy. In light of these findings, we reviewed the role of viral infection–induced inflammatory response processes and autoimmunity in the pathogenesis of KFD. Simultaneously, we explored the common pathogenic features of KFD and SLE, while also performing a differential diagnosis to distinguish between the two conditions.

## Case description

An 18-year-old Chinese girl was admitted to our hospital with a 1-month duration fever (38–40°C) and bilateral cervical lymphadenopathy. Her first fever occurred after catching a cold, with a maximum body temperature of 38.5 °C accompanied by a sore throat, and she was initially treated at a clinic with azithromycin and antipyretics for 1 day, with resolution of the fever for a week. However, 1 week later, she still had recurrent fever, with a maximum body temperature of 39.6 °C; she was treated at home with oral acetaminophen to reduce the temperature. Initially, antipyretic drugs were effective, and her body temperature dropped to 37°C. On the 20th day of fever, she re-commenced having multiple daily fevers of up to 40°C, and oral antipyretic medication was ineffective. Meanwhile, she developed swelling and pain in the neck lymph nodes, and routine blood examination laboratory tests at the local hospital showed a white blood cell count of 2.92 ×10^9^/L (3.5–9.5×10^9^/L) and a C-reactive protein level of 4.29 mg/L (0–8.0 mg/L). An ultrasound showed bilateral parotid glands, bilateral neck, and bilateral submandibular lymph nodes with mild enlargement. Antiviral treatments in the local hospital were not effective. She continued to experience recurrent fever with progressive enlargement of the lymphadenopathies and was then referred to our department for further treatment.

On the first examination after admission to our department, the patient was febrile and had painful lymphadenopathy in the right side of the neck; she also had rashes on the face and arthralgia of the metacarpophalangeal joint of the right hand. Notably, the rashes observed on both sides of the face were asymmetric, discrete, and non-scaly, without photosensitivity. She denied weight loss or exhaustion. There was no significant high-risk sexual, travel, or past medical or family illness history. Physical examination showed multiple enlarged palpable lymph nodes in the neck, with the largest one located in the right cervical, which was round and tough, tender, and exhibited clear boundaries, no adhesion to the surrounding tissue, and no hepatomegaly or splenomegaly.

**Table 1** presents the laboratory test results of the patient, with the normal range indicated. A lymph node ultrasound showed multiple enlarged lymph nodes in the bilateral neck and right axilla, with the largest node located in the right cervical upper jugular II, measuring 34×10 mm.

**Table 1:** Laboratory data.

Variable	Reference range	Pre-admission	On admission	Post-discharge
White blood cell count (×10^9^/L)	3.5–9.5	2.92	1.72	4.84
Neutrophil (×10^9^/L)	1.8–8.3	0.83	0.87	2.02
Lymphocyte (×10^9^/L)	1.1–3.2	1.05	0.7	2.28
Eosinophil (×10^9^/L)	0.02–0.52	0.01	0.0	0.19
C-reactive protein (mg/L)	0–8.0	4.29	6.31	
Red blood cell sedimentation rate (mm/h)	0–15.0		88.0	
Serum ferritin (ng/ml)	12–135		199.78	
Serum amyloid A (mg/L)	<10		64.02	
Procalcitonin (ng/ml)	0–0.5		0.06	
Lactate dehydrogenase (U/L)	120–250		341	
Interleukin-6, IL-6 (pg/mL)	≤5.3		29.88	
Interferon-γ, INF-γ (pg/mL)	≤7.42		11.19	
Alanine aminotransferase, ALT (U/L)	6–29		43	26
Aspartate aminotransferase, AST (U/L)	10–31		66	16
Peripheral blood lymphocyte subsets				
Lymphocyte (/μL)	1,760–3,000		495	
Total T-lymph (/μL)	1,169–2,071		374	
CD4+ T cell (/μL)	554–1,109		194	
CD8+ T cell (/μL),	423–900		151	
B-lymphocyte (/μL)	176.56–415.00		91	
NK-cell (/μL)	232–789		31	
Antinuclear antibody profile and immunoglobulin, complement				
Anti-nuclear antibody titer	(1: <100)		1:100-	1:100-
Anti-double-stranded DNA, anti-dsDNA (IU/ml)	<100		<10	<10
Anti-Smith, anti-Sm	negative (–)		negative (-)	negative (-)
Anti-Ro, SSA	negative (-)		negative (-)	negative (-)
Anti-La, SSB	negative (-)		negative (-)	negative (-)
Immunoglobulin G (g/L)	7.00–16.00		17.6	
Complement factor 3 (g/L)	0.90–1.80		1.21	
Complement factor 4 (g/L)	0.100–0.400		0.452	
Virus Antibody Measurement				
EBV viral capsid antigen IgG (U/ml)	≤20		116	
EBNA-1 IgG (U/ml)	≤5–20		579	
EBV viral capsid antigen IgM (U/ml)	≤20–40		4.16	
CMV IgM (S/CO)	≤0.7–1.0		0.801	
CMV IgG (U/ml)	≤0.5–1.0		68.730	
HIV antigen-antibody combination test (S/CO)	<1.0		0.09	
HBsAg(IU/ml)	<0.05		0.0	
HSV-1 IgG (S/CO)	≤0.6–1.0		0.019	
HSV-2 IgG (S/CO)	≤0.5–1.0		0.062	
Tuberculosis				
Tuberculin intradermal reaction	negative (-)		negative (-)	
Tuberculosis antibody	negative (-)		negative (-)	
T-SPOT	negative (-)		negative (-)	

EBV, Epstein–Barr virus; CMV, cytomegalovirus; HSV, human herpes simplex virus.

**Figure 1:**
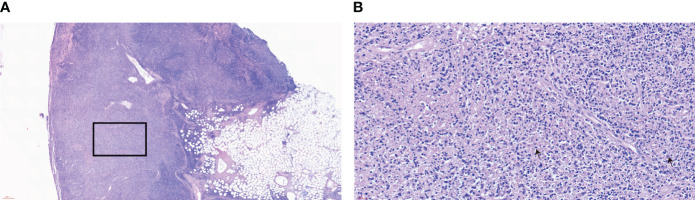
Lymph node biopsy pathology **(A)** HE×40, **(B)** HE×400. The stains of the patient’s cervical lymph nodes show the characteristic features of histiocytic necrotizing lymphadenitis. Lymphoid follicular hyperplasia is observed, and the structure of the lymph node in the light-stained area is disrupted. Under a high-power view, histiocytic infiltrate with karyorrhectic debris is seen, along with partly histiocytic crescent-shaped nuclei and large immunoblastic cells at the edges of necrotic foci. The box shows a tissue necrosis lesion. Arrows indicate characteristic crescent-shaped nuclei.

**Figure 2:**
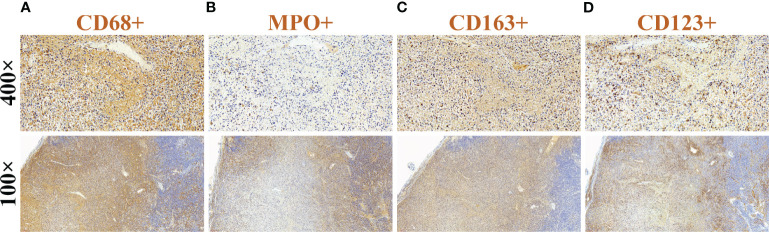
Immunohistochemical findings of cervical lymph nodes. **(A)** CD68(+), **(B)** MPO(+), **(C)** CD163(+), and **(D)** CD123(+).

Combining the patient’s medical history and examination results, Kikuchi-Fujimoto disease was suspected, and on day 35 of fever, she underwent a right-sided cervical lymph node excisional biopsy. The postoperative pathology showed numerous lympho-histiocytic cells, multiple necrotic areas with marked karyorrhexis and proliferation of distinctive crescentic histiocytes, and a few remaining lymphoid follicles in the surrounding area without neutrophils or eosinophils (**Figure 1**). Immunohistochemistry was positive for lysozyme, CD68, MPO, CD163 and CD123 (**Figure 2**). The diagnosis of KFD was confirmed based on these pathologic manifestations. After being diagnosed with KFD, we administered 20 mg/d of prednisolone to the patient, after which the patient’s general condition improved. On the first day of prednisolone treatment, the patient’s temperature dropped to normal and the lymph node pain disappeared. Her arthralgia disappeared as the fever subsided. By the third day of medication, a physical examination revealed a noticeable reduction in the size of the patient’s enlarged lymph nodes. The treatment plan involved starting with 20 mg of prednisolone daily and then reducing the dosage by 5 mg per week until discontinuation. Eventually, she received a total of 1 month of corticosteroid hormone therapy.

After discontinuing the hormone therapy for 1 month, the patient went to the hospital for a follow-up examination, and the results showed that on examination, no enlarged lymph nodes were palpable in the neck and her facial rash had disappeared. Furthermore, there was no fever, lymph node swelling, or arthralgia. Liver function: ALT 26 U/L (6–29 U/L), AST 16 U/L (10–31 U/L). White blood cell count: 4.84×10^9^/L (3.5–9.5×10^9^/L), neutrophil count: 2.02×10^9^/L (1.8–8.3×10^9^/L), and eosinophil count: 0.19×10^9^/L (0.02–0.52×10^9^/L), all within normal ranges. ANA, dsDNA, SSA, and SSB were negative. At present, the patient has been followed up for 6 months and has no symptoms of recurrence. ANA, dsDNA, SSA, and SSB are still negative; however, the patient should still be followed up for a long time to be alert to the occurrence of SLE.

## Discussion

Kikuchi-Fujimoto disease is a self-limiting disease that is prevalent in young women. KFD is primarily prevalent in populations from Asia; however, it has been reported worldwide. Kucukardal et al. (12) analyzed 330 cases of KFD, with approximately 55% from Asia, 27% from Europe, 7% from North America, and 1.8% from South America. The lymph node tissue in KFD shows focal necrosis of paracortical histiocytes that is characteristically devoid of granulocytes, and the necrotic area is surrounded by T-cell immunoblasts, characteristic crescentic histiocytes with cellular debris, and plasmacytoid dendritic cells (PDCs) (13, 14). The clinical manifestations and pathologic findings may suggest a viral etiology. Related viruses include the Epstein–Barr virus and cytomegalovirus, human papillomavirus, parvovirus B19, hepatitis B virus, human T-lymphotropic virus 1, Torque teno virus/Torque teno-like mini virus, HIV, and parainfluenza virus (6, 15, 16). In this case, the patient was positive for IgM and IgG of CMV and positive for IgG of EBV, suggesting current CMV infection and previous EBV infection. Therefore, we consider that the CMV infection in this patient may be a triggering factor for KFD. The previous EBV infection should not be ignored. This phenomenon of previous EBV infections leading to autoimmune diseases has been found in studies on EBV-induced SLE, which is currently explained through gene environment interactions at the molecular level (17). KFD and SLE exhibit notable similarities in their pathophysiologies, and some research reports suggest that the incidence of KFD merging or developing into SLE is 1.3–7% (18). Meanwhile, it is worth noting that EBV is involved in SLE pathogenesis (17) and that CMV aggravates the autoimmune phenomenon in SLE (19). Therefore, it is very important to identify KFD and SLE.

Viral infections cause changes in the number and function of CD4+ T cells and CD8+ T cells in the immune system. By reviewing the literature, we found that it was reported that there was a large number of T cells in the lesions of patients with KFD, and there were more CD8+ cells than CD4+ cells in immunohistochemistry (1, 20–23), which was characterized by blastic transformation of CD8+ cells and apoptosis of CD4+ cells (1, 24). However, the clinical course of the disease and the ratio of CD4+/CD8+ cells in the lymph nodes did not correlate with peripheral blood (1). Moreover, the number of CD8+ cells and PDCs within the lesions tended to increase with time, and the number of CD4+ cells within the lymph node lesions decreased sharply at weeks 2~4 and then tended to increase whereas, in the non-lesioned tissues, the number of CD4+ cells gradually decreased with time (11). Furthermore, CD4+ T cells can differentiate into different subpopulations, of which the five main subpopulations are Th1, Th2, Th17, Treg (T-regulatory), and Tfh (follicular T-helper) cells, which fight different types of infections and regulate immune homeostasis (25). Among them, Th17 (26), Treg (27), Tfh (21), and Th9 (28) cells have been shown to be involved in the development of autoimmune diseases. In SLE, there is a decrease in the quantitative and/or qualitative defects of Treg cells (29), while the number of Th17 cells and Th17-related cytokines (such as IL-17 and IL-6) increases (30, 31). Tfh cells also secrete an important cytokine, IL-21, which is essential for maintaining CD8+ T-cell function during chronic virus-induced inflammation (32, 33). In other words, changes in CD4+ T cells affect not only the secretion of cytokines such as IL-17, IL-6, and IL-21, but also the function of CD8+ T cells, and, in combination with changes in the number of CD4+ T cells in the lesions of KFD, may be one of the reasons for the possibility of developing into autoimmune diseases like SLE. However, their specific pathogeneses remain unclear.

In addition, elevated interferon levels have been found in patients with KFD (34–36). Li et al. (36) suggested that Kikuchi-Fujimoto disease is mediated by an aberrant type I interferon (IFN) response, with significant upregulation of IFN-related genes and an increase in CD123+ plasmacytoid dendritic cells, which are myeloid cells that secrete IFN in response to viral infection. Type I IFN can be divided into two types: IFN-α and IFN-β (37). Type I IFN enhances innate immunity by stimulating the differentiation and maturation of dendritic cells and the function of natural killer cells and enhances adaptive immunity by promoting the activation and differentiation of T and B cells and the development of immune memory (35). In KFD, Sato et al. (11) proposed the hypothesis that interferon IFN-α production in response to viral infection induces the transformation of CD8+ cells, CD4+ cells, and monocytes into immunoblasts, apoptotic cells, and macrophages, respectively, and that apoptotic cells are subsequently phagocytized by macrophages. There is experimental evidence that the use of specific receptor bindings of PDCs to reduce the expression of type I INF improves the skin manifestations of SLE and reduces the expression of IFN-responsive genes in the bloodstream (38). Additionally, Munroe et al. (39) found that the enhancement of the type II IFN (IFN-γ) pathway permitted the accumulation of autoantibodies and the subsequent elevation of IFN-α activity. The pathologic tissue of our patient showed elevated PDCs as well as elevated IFN-γ in the peripheral blood, which suggests that the interferon system may be involved in the development of disease. This observation reaffirms the shared pathogenic features between KFD and SLE.

**Figure 3:**
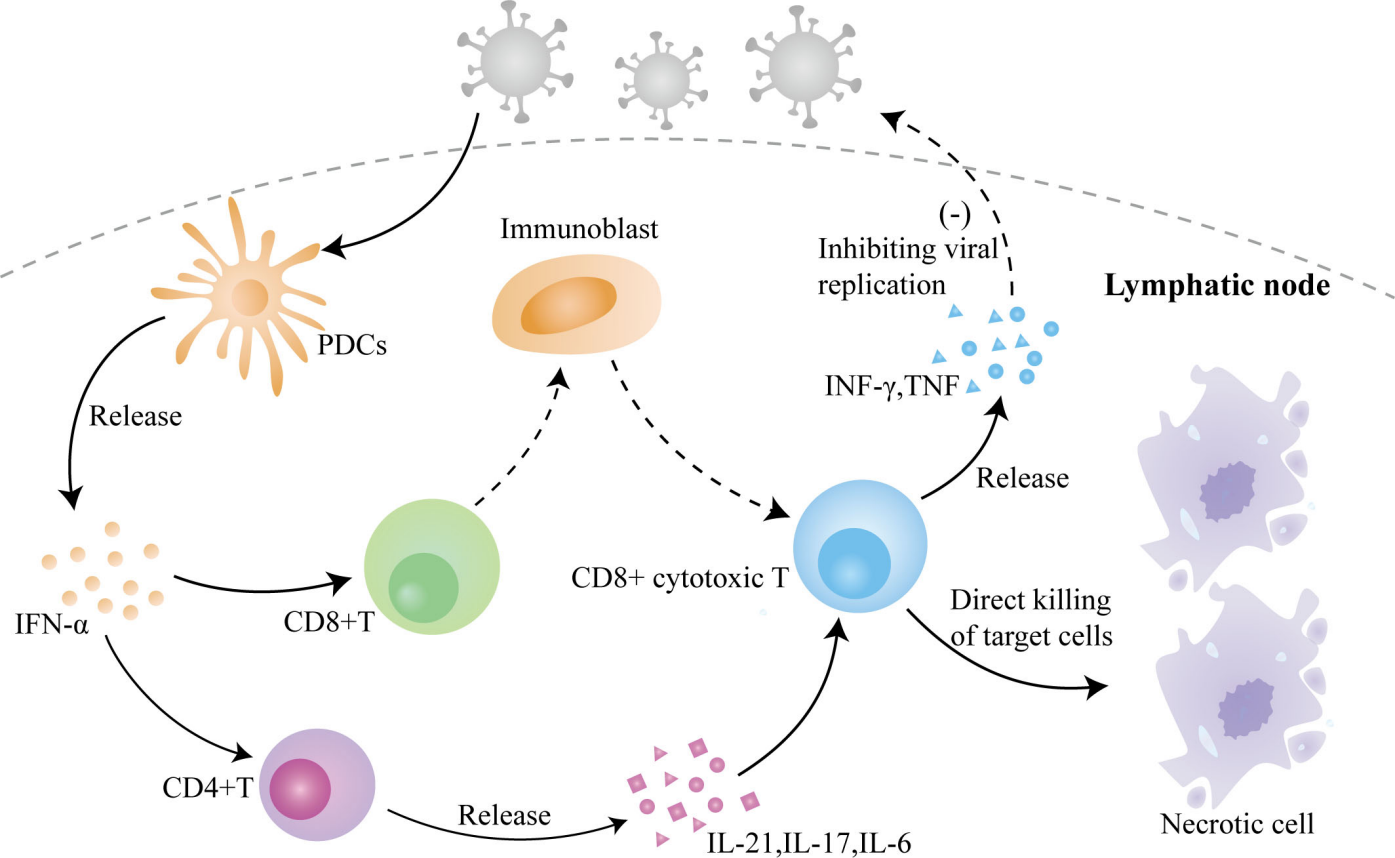
The role of viral infection–induced inflammation in the pathogenesis of KFD. Invasion of the virus causes the activation of PDCs to release IFN-α, which activates CD4+ T cells and CD8+ T cells, causing differentiation and maturation of CD4+ T cells and the release of cytokines and activation of CD8+ T cells, which leads to a massive proliferation of immunoblasts and their differentiation into CD8+ cytotoxic T lymphocytes to directly kill the target cells, with the simultaneous release of cytokines to inhibit viral replication.

In summary, KFD is a self-limiting autoimmune disease caused by viral infection (**Figure 3**), with fever and lymph node enlargement as the main clinical manifestations. The similarity of its pathogenesis and its pathologic manifestations to SLE complicates its diagnosis, and the fact that patients with this disease may combine or develop SLE due to this common pathogenesis makes it important to follow up this disease. Our patient’s initial symptoms were fever as well as lymph node enlargement and necrosis, and she had no manifestations of lupus nephritis or neuropsychiatric involvement, except for atypical facial rash and arthralgia; thus, she did not align with the typical features of SLE. Furthermore, tests for ANA, ds-DNA, SSA, and SSB antibodies all yielded negative results and there was no indication of low C3 or C4, further reducing the likelihood of SLE. However, it is essential to recognize that juvenile-onset SLE often exhibits atypical clinical symptoms and significant variations in its presentation (40). Even when patients lack classical symptoms, the possibility of SLE should be considered and a high level of vigilance is warranted. At present, the patient remains under ongoing follow-up to monitor her condition.

## Data availability statement

The original contributions presented in the study are included in the article/supplementary material. Further inquiries can be directed to the corresponding author.

## Ethics statement

The studies involving humans were approved by the Ethics Committee of the First Affiliated Hospital of Dalian Medical University. The studies were conducted in accordance with the local legislation and institutional requirements. The participants provided their written informed consent to participate in this study. Written informed consent was obtained from the individual(s) for the publication of any potentially identifiable images or data included in this article.

## Author contributions

JLY: Writing – original draft, Writing – review & editing, Conceptualization, Methodology. ZL: Writing – original draft, Writing – review & editing, Visualization, Investigation. BZ: Writing – review & editing, Visualization. YNH: Writing – review & editing. TYZ: Writing – review & editing, Conceptualization, Supervision.
